# Metabolic connectivity alterations in amyotrophic lateral sclerosis: Individual network analysis based on Wasserstein distances

**DOI:** 10.1162/IMAG.a.1343

**Published:** 2026-08-20

**Authors:** Chunmeng Tang, Joke De Vocht, Philip Van Damme, Koen Van Laere, Michel Koole

**Affiliations:** Nuclear Medicine and Molecular Imaging, Imaging and Pathology, KU Leuven, Leuven, Belgium; Laboratory of Neurobiology (KU Leuven), Department of Neurosciences, Leuven Brain Institute (LBI), KU Leuven, Leuven, Belgium; Neurology department, University Hospitals Leuven, Leuven, Belgium; Division of Nuclear Medicine, University Hospitals Leuven, Leuven, Belgium

**Keywords:** metabolic connectivity, individual brain network, Wasserstein distance, brain FDG PET, amyotrophic lateral sclerosis (ALS)

## Abstract

Molecular connectivity analysis with positron emission tomography (PET) imaging offers a promising approach for characterising brain network alterations in neurodegenerative disorders. In this study, we introduce Wasserstein distance (WD) as an alternative to Kullback–Leibler divergence similarity estimation (KLSE) for constructing single-subject metabolic connectivity networks. Using ^18^F-FDG PET data from 167 individuals with amyotrophic lateral sclerosis (ALS), 36 healthy volunteers (HV), and 25 ALS mimics, we generated WD- and KLSE-based connectivity matrices across 77 atlas-defined brain regions and evaluated corresponding graph theory-based nodal metrics. WD- and KLSE-derived nodal measures were strongly correlated, indicating methodological consistency. Compared with HVs, age-matched subjects in the ALS group (ALS_amHV_) showed significant alterations in frontal, temporal, cerebellar, and occipital network nodes, with WD-based metrics revealing differences across more brain regions than the KLSE-based approach. Support vector machine classification of ALS_amHV_ vs. HV demonstrated that both connectivity approaches matched voxel-wise PET performance with accuracy higher than 0.80 (yet significantly lower than voxel-wise data, p<0.05 ), while significantly outperforming image-based classification for ALS vs. ALS mimics (p<0.01 ), with WD achieving the highest accuracy of 0.65 (p<0.001 ). These findings support WD-based metabolic connectivity as a sensitive, data-efficient framework for detecting disease-related network alterations and motivate its application to a broader range of PET tracers and cohorts.

## 1 Introduction

Positron emission tomography (PET) is a well-established modality for generating imaging biomarkers of central nervous system (CNS) disorders, supporting diagnosis, monitoring therapeutic response, and assessing dose-dependent drug–target engagement. However, conventional univariate PET analyses are limited in their capacity to capture interactions among spatially distributed neuronal populations and, therefore, do not fully characterise the brain’s connectome. Network neuroscience, an expanding discipline based on graph theory, offers a rigorous framework for studying the topology of complex brain networks at both local and global scales (Bassett & Sporns, 2017; Bullmore & Sporns, 2009; Rubinov & Sporns, 2010; Sporns et al., 2004; Stam et al., 2009; Stam & Reijneveld, 2007). Within this framework, the brain is modelled as a graph consisting of nodes, defined by atlas-based volumes of interest (VOIs), and corresponding edges that reflect the strength of interregional associations. The resulting network architecture can be quantitatively assessed using a wide range of graph metrics that can differentiate patient groups and may serve as diagnostic imaging markers. Traditionally, such brain networks are constructed from structural and functional connectivity (SC and FC), typically derived from diffusion-weighted MRI and resting-state BOLD fMRI time series, respectively (Buckner et al., 2013). More recently, methodological advances have expanded network neuroscience into the field of molecular connectivity, wherein PET-based approaches capture pathway-specific neuronal interactions with potential high molecular specificity, supported by a broad range of tracers for neural activity, neurotransmission, and proteinopathies (Sala et al., 2023).

In PET, molecular connectivity is commonly inferred from interregional covariance or dependency patterns in tracer uptake across subjects. A variety of methodological frameworks have been proposed to construct such PET-based molecular connectivity networks. Among early and widely used approaches, regional correlation analyses assess the interregional coupling of tracer uptake across subjects and have been, for example, applied to the study of neurodegenerative diseases using ^18^F-FDG PET (Catricalà et al., 2020; Q. Huang et al., 2019; Huber et al., 2020). In addition, multivariate decomposition techniques, such as scaled subprofile model principal component analysis (SSM-PCA), have been extensively employed to identify disease-related metabolic covariance patterns, with applications in Parkinson’s disease and dementia with Lewy bodies (Z. Huang et al., 2020; Meles et al., 2020; Peng et al., 2021; Rodriguez-Rojas et al., 2019; Rus et al., 2020; Stockbauer et al., 2023). Similarly, independent component analysis (ICA) has been used to extract spatially independent metabolic networks and investigate alterations in network organisation across neurological conditions (Li et al., 2020; Ripp et al., 2020). Another commonly used method, among others, sparse inverse covariance estimation (SICE), also referred to as Gaussian graphical modelling (Friedman et al., 2008), has been applied, for instance, to estimate conditional dependencies between brain regions and quantify multiscale alterations in metabolic connectivity in early Parkinson’s disease (Sala et al., 2017) and to characterise metabolic network changes and identify relevant biomarkers in Alzheimer’s disease (S. Huang et al., 2010), both using ^18^F-FDG PET, among other applications. However, these approaches are inherently group based, which limits their direct applicability for individual-level molecular connectivity assessment. Developing reliable single-subject measures of molecular connectivity, therefore, remains essential in neurodegenerative research (Sala & Perani, 2019), to allow diagnostic and prognostic biomarker development and to assess therapeutic effects in emerging interventions on an individual, patient-specific level (Fortier et al., 2019).

To address this limitation, recent work has shifted towards molecular connectivity estimation at the single-subject level. One proposed method derives individual metabolic connectivity by integrating a global matrix, computed from mean tracer uptake in healthy volunteers, with a weighting matrix defined by patient-specific z-scores relative to the healthy volunteer distribution (S.-Y. Huang et al., 2020). Another approach employs Kullback–Leibler divergence similarity estimation (KLSE) between local probability density functions (PDFs), which has been shown to predict progression from mild cognitive impairment (MCI) to Alzheimer’s dementia (AD) (Wang et al., 2020). The KLSE framework has also been applied to characterise the effects of healthy ageing on brain metabolic networks using ^18^F-FDG PET (Mertens et al., 2022), and to construct individual metabolic connectomes in conditions such as temporal lobe epilepsy and evaluate their association with surgical prognosis (Y. Tang et al., 2024).

In parallel to KLSE-based applications, there is growing interest in complementary approaches beyond KLSE that can directly quantify distances between regional tracer uptake distributions at the individual level. In this context, Wasserstein distances, derived from optimal transport theory and often referred to as the “Earth Mover’s Distance” (Givens & Shortt, 1984; Vaserstein, 1969), offer an approach for computing distances between regional distributions directly from voxel-wise data without requiring matched value ranges or substantial overlap between probability density functions (PDFs) (Tuan et al., 2026). Unlike KLSE, which is sensitive only to the overlapping parts of estimated probability density functions and can result in infinite divergence or zero similarity for non-overlapping distributions regardless of the magnitude of the difference, the Wasserstein distance remains well defined in such cases. Moreover, it captures global distributional differences without requiring probability density function estimation, as it can be computed directly from voxel-wise uptake values.

Having established PET-based frameworks for estimating molecular connectivity at the individual level, the potential utility of these approaches can be evaluated in neurodegenerative diseases, including amyotrophic lateral sclerosis (ALS). ALS is a progressive, heterogeneous neurodegenerative disorder that is typically fatal within 2–5 years of symptom onset (van Es et al., 2017). It involves degeneration of upper and lower motor neurons in the motor cortex, brainstem, and spinal cord (Masrori & Van Damme, 2020), leading to progressive weakness and paralysis, with respiratory failure being the most common cause of death (van Es et al., 2017). Previous neuroimaging studies have demonstrated that ^18^F-FDG PET is a reliable diagnostic marker in ALS, consistently revealing peri-rolandic and variably prefrontal hypometabolism in affected individuals (Pagani et al., 2014; Van Laere et al., 2014). In addition, ^18^F-FDG PET has been proposed as a marker of disease severity, with metabolic alterations correlating with cognitive impairment, comorbid frontotemporal dementia (FTD), and prognosis (Foucher et al., 2024; Van Weehaeghe et al., 2016). Collectively, these studies report high accuracy in distinguishing persons with ALS from healthy volunteers using ^18^F-FDG PET, with classification accuracies of approximately 0.86 achieved using linear support vector machine classifiers (C. Tang et al., 2026).

However, despite the reported diagnostic value of ^18^F-FDG PET for distinguishing ALS from healthy volunteers, differentiating ALS from clinically relevant ALS-mimicking diseases remains challenging. Persons with ALS and those with ALS mimics can exhibit largely similar patterns of brain glucose metabolism, resulting in low prediction accuracy, only subtle VOI-level differences that do not survive correction for multiple comparisons, and an absence of significant voxel-wise clusters. This likely reflects the clinical and biological heterogeneity of ALS mimics, as well as their potential resemblance to early ALS manifestations, which together complicate differential diagnosis.

Building on previous work using FDG-PET–derived metabolic connectivity, this study evaluated metabolic network analyses based on Wasserstein distance and KLSE. We assessed performance in two clinically relevant contrasts, distinguishing ALS from healthy volunteers, a relatively straightforward classification with high reported accuracy, and distinguishing ALS from ALS mimics, a substantially more challenging diagnostic scenario. By comparing network-derived metrics and classification performance across these contrasts, we aimed to quantify the added value of Wasserstein distance for single-subject metabolic connectivity analysis in ALS.

## 2 Materials and Methods

### 2.1 Study cohorts

^18^F-FDG PET data of 36 healthy volunteers (HV, 55.0 ± 13.0 years, 17M/19F), 25 persons with an ALS-mimicking disease (ALS mimics, 62.4 ± 8.0 years, 17M/8F, final diagnoses is given in Supplementary Table S1), and 167 persons diagnosed with ALS (63.9 ± 11.1 years, 103M/64F) from a previous study were included (De Vocht et al., 2023; C. Tang et al., 2026). ^18^F-FDG PET data acquired with a single PET/CT scanner between May 2012 and April 2023 were considered to avoid any bias in the group comparison due to variations in image quality across different PET/CT systems.

The dataset was collected at the Neuromuscular Reference Center (NMRC) at UZ Leuven and represents a Flemish population of PwALS and healthy volunteers. Data collection was approved by the local ethics committee of UZ Leuven and was in compliance with the Declaration of Helsinki. Written informed consent of all subjects was obtained.

### 2.2 Brain ^18^F-FDG PET imaging

^18^F-FDG PET scans were acquired with a Siemens Biograph 40 “TruePoint” PET/CT using a 5–20 minutes list mode scan protocol, starting 30 minutes after injection. During the acquisition, the patient’s head was positioned in a head holder and immobilised with a vacuum pillow. Before the PET scan, a low-dose CT scan was acquired to generate a CT-based attenuation map. PET data were corrected for attenuation, scatter, randoms, deadtime, and decay, and reconstructed using ordered-subset expectation maximisation (OSEM) with 3 iterations and 21 subsets. PET reconstructions included point spread function (PSF) modelling and post-smoothing with a 2 mm FWHM (full width half maximum) Gaussian kernel, resulting in a PET image resolution of approximately 6 mm.

### 2.3 Single-subject brain metabolic connectivity based on Wasserstein distance and KLSE

We constructed single-subject metabolic connectivity networks using Wasserstein distance (WD) and Kullback–Leibler divergence-based similarity estimation (KLSE). Brain metabolic connectivity was modelled as a weighted, undirected graph (network), excluding self-connections, in which nodes represent cortical and subcortical regions and edge weights reflect pairwise (dis)similarity in regional tracer distributions. Network nodes were defined using a modified version of the Hammers N30R95 grey matter atlas (Faillenot et al., 2017; Gousias et al., 2008; Hammers et al., 2003), excluding regions containing fewer than 100 voxels (corresponding to 800 mm^3^) and their corresponding contralateral counterparts to avoid unstable distributional estimates due to small region size and partial-volume effects, resulting in a total of 77 regions included in the analysis. Corresponding region volumes are given in Supplementary Table S2).

For each subject, the brain ^18^F-FDG PET image was spatially normalised to Montreal Neurological Institute (MNI) space using an FDG PET template (SPM12; Wellcome Trust Centre for Neuroimaging). The image was masked to the brain regions of interest, and voxel intensities within the mask were converted to z-scores using the within-mask mean and standard deviation computed per subject. Each atlas region was considered as a node in the metabolic network.

The pairwise regional Wasserstein distance $D_{X,Y}^{\text{WD}}$ between brain regions *X* and *Y* was computed from the voxel-wise z-score distributions using the SciPy package (v1.13.1, Python v3.9.23). Given that regions *X* and *Y* are represented by voxel-wise z-scores ${\left\{x_{i}\right\}}_{i=1}^{n}$ and ${\left\{y_{j}\right\}}_{j=1}^{m},$ respectively, each voxel’s z-score is considered as a sample with equal weight in the distribution. The optimal transport plan is monotonic, allowing $D_{X,Y}^{\text{WD}}$ to be computed exactly from sorted voxel-wise z-scores without estimation of probability density functions, avoiding histogram binning or kernel density estimation that would otherwise be required for KLSE-based approaches. The $D_{X,Y}^{\text{WD}}$ were then transformed using the exponential mapping with scaling parameter γ = 3 as described in Eq. (1), where *A*_*X*, *Y*_ denotes the edge weight between regions *X* and *Y* in adjacency or connectivity matrix of the metabolic connectivity network and $D_{X,Y}^{\text{WD}}$ the corresponding pairwise regional Wasserstein distance between regions *X* and *Y*. This inverse exponential operation transforms $D_{X,Y}^{\text{WD}}$ into a bounded similarity measure, ensuring smooth down weighting of larger distances without introducing hard thresholds and maintaining numerical stability across subjects. The computation of the Wasserstein distance is summarised in the following algorithm. Given the 77 brain regions included in the analysis, this resulted in a 77 × 77 adjacency matrix for each subject.

**Table:** 

**1D Wasserstein-1 distance** (uniform weights; unequal sample sizes allowed, *n* ≠ *m*)
**Input:** Sample sets $X={\left\{x_{i}\right\}}_{i=1}^{n}$ and $Y={\left\{y_{j}\right\}}_{j=1}^{m}$ **Output:** $D_{X,Y}^{\text{WD}}$ Sort *X* in ascending order to obtain $x_{1}\leq \cdots \leq x_{n}$; Sort *Y* in ascending order to obtain $y_{1}\leq \cdots \leq y_{m}$; *i*←1, *j*←1; $r_{x}\leftarrow 1/n$ , $r_{y}\leftarrow 1/m$ ; $D_{X,Y}^{\text{WD}}\leftarrow 0$ ; **while** *i * ≤ * n* and *j * ≤* m* **do** $\delta \leftarrow \text{min}\left(r_{x},r_{y}\right)$**;** $D_{X,Y}^{\text{WD}}\leftarrow D_{X,Y}^{\text{WD}}+\delta \;\;\cdot \;\;\left|x_{i}-y_{j}\right|$; $r_{x}\leftarrow r_{x}-\delta$ ; $r_{y}\leftarrow r_{y}-\delta$ ; **if** *r_x_* = 0 **then** i←i+1 ; **if** *i *≤ *n* **then** $r_{x}\leftarrow 1/n$ ; **end** **end** **if** *r_y_* = 0 **then** j←j+1 ; **if** *j * ≤ * m* **then** $r_{y}\leftarrow 1/m$ ; **end** **end** **end** **return** $D_{X,Y}^{\text{WD}}$ ;

$$A_{X,Y}=e^{-\gamma D\begin{array}{c} WD2\\ X,Y \end{array}}.$$ (1)

For the KLSE-based approach, regional probability density functions (PDFs) were estimated using kernel density estimation (KDE) with optimal bandwidth selection based on the diffusion method (Botev et al., 2010), as implemented in the KDE-diffusion toolbox for Python. A symmetric Kullback–Leibler divergence between the PDFs of regions *X* and *Y*, denoted *P_X_*(*z*) and *P_Y_*(*z*), respectively, was then computed according to Eq. (2).

$$D_{X,Y}^{\text{KL}}=\int_{z}\left(P_{X}\left(z\right)\text{log}\frac{P_{X}\left(z\right)}{P_{Y}\left(z\right)}+P_{Y}\left(z\right)\text{log}\frac{P_{Y}\left(z\right)}{P_{X}\left(z\right)}\right)dz.$$ (2)

This divergence measure was subsequently transformed by the exponential mapping with γ = 1 (Eq. (1)) to the corresponding edge weight *A*_*X*, *Y*_ between regions *X* and *Y* in the adjacency matrix of the metabolic connectivity network.

### 2.4 Graph theory network metrics

To compare the network topology obtained with both approaches, we quantified graph theory-based nodal metrics derived from the adjacency matrices of the resulting metabolic connectivity networks. For both WD- and KLSE-based metabolic networks, nodal metrics were computed using the Brain Connectivity Toolbox for Python (bctpy, v0.6.1), a Python implementation of the original MATLAB toolbox (Rubinov & Sporns, 2010). For each subject, node strength, average path length, clustering coefficient, local efficiency, and betweenness centrality were computed for all 77 regions. These regional metrics were then analysed as left–right pairs except for the brainstem, as well as through the corresponding asymmetry index (AI), which is defined as $\frac{R-L}{R+L}$ .

### 2.5 Metabolic connectivity differences in ALS versus healthy volunteers and ALS versus ALS mimics

To minimise potential age-related confounding effects, groups with significantly different age distributions were age matched by selecting subjects from the larger group using a nearest-neighbour algorithm based on age.

After age matching, linear support vector machines (SVMs) were trained and tested using 10-fold cross-validation (scikit-learn v1.4.2) on the resulting adjacency matrices to classify ALS versus healthy volunteers and ALS versus ALS mimics. Classification performance based on the WD- and KLSE-derived adjacency matrices was compared with an image-based SVM approach, in which the normalised voxel values from the ^18^F-FDG PET images were used as training features.

For each atlas region, nodal metrics in the left and right hemispheres, as well as their asymmetry indices, were compared between ALS and healthy volunteers and between ALS and ALS mimics for both the WD- and KLSE-based methods. Finally, correlations between nodal metrics derived from the WD-based metabolic network in the healthy volunteer group and the corresponding metrics of the KLSE-based metabolic network were assessed.

### 2.6 Statistical analysis

Group differences in connectivity were visualised as maps of standardised mean differences (Cohen’s *d*) for the healthy volunteer (HV) versus age-matched ALS (ALS_amHV_) groups and for the ALS versus ALS mimics group. This provided a quantitative, scale-independent measure of effect size for both the WD- and KLSE-based connectivity matrices.

To assess whether these group-level differences translated into discriminative information at the individual level, SVM classifiers using the connectivity matrices and voxel-wise PET data as features were evaluated for differentiating the same group pairs, in terms of average accuracy, sensitivity, specificity, and area under the receiver operating characteristic curve (ROC-AUC). In line with our previous voxel-wise SVM implementation, a soft-margin linear SVM was used, implemented in Python (v3.9; scikit-learn v1.4.2) with squared hinge loss and *L*_2_ regularisation, while balanced class weights were applied where appropriate. For voxel-wise PET classification, reconstructed PET images were spatially normalised to MNI space, masked to include grey and white matter, flattened into 1-dimensional feature vectors, and normalised to unit *L*_2_-norm before classification. Hyperparameters for the WD-, KLSE-, and voxel-wise PET-based classifiers are summarised in the Supplementary Table S3 and were selected on a small stratified subset of the data prior to the cross-validation folds. Therefore, nested cross-validation was not used. In addition, the training and testing folds were stratified and consistent across methods to make the evaluation of the full classification procedure more transparent. Performance metrics were reported as the mean and standard deviation across 10 repetitions of 10-fold cross-validation with reshuffled datasets and were compared across WD- and KLSE-based connectivity approaches as well as voxel-wise methods using paired one-sided Welch’s *t*-tests with a significance threshold of p<0.05 .

As a complementary assessment to the group differences, the correspondence between WD- and KLSE-based connectivity estimates was quantified. Specifically, Pearson’s correlation coefficient (*r*) was computed between corresponding edges of the WD- and KLSE-based connectivity matrices at the subject level within the HV group. In addition, correspondence between network nodal metrics derived from the two methods was assessed across the different groups using Pearson’s *r* and associated *p*-values. Nodal metrics were summarised as group means and standard deviations, and boxplots were used for visual comparison. Group differences in nodal metrics were assessed using two-sided Welch’s *t*-tests. For the regional statistical analyses, Benjamini–Hochberg false discovery rate (BH-FDR) correction was applied to all the regional tests within each metric separately, and not applied jointly across all methods and metrics combined. Results were considered significant at FDR-adjusted q<0.05 (Benjamini & Hochberg, 1995).

## 3 Results

### 3.1 Study cohorts

Table 1 summarises the age information of the HV, ALS, and ALS mimics cohorts. As a significant age difference was observed between the HV and ALS groups (8.9 ± 2.4 years, p<0.005 ), the ALS group was age matched to the HV group (ALS_amHV_) using a nearest-neighbour algorithm.

**Table 1.** Age characteristics of the healthy volunteer (HV), ALS, and ALS mimics cohorts.

HV	ALS	ALS_amHV_*	ALS mimics
*N* = 36 [55.0±13.0]	*N* = 167 [63.9±11.1]	*N* = 72 [56.7±11.1]	*N* = 25 [62.4±8.0]

For comparisons with the HV group, the ALS cohort was age matched (ALS_amHV_). The number of subjects (*N*) is presented as well as the age (years) in square brackets as mean ± SD.

* ALS group was matched to the HV group by age using a nearest-neighbour algorithm.

### 3.2 Metabolic connectivity adjacency matrices in ALS_amHV_ versus healthy volunteers and ALS versus ALS mimics

**Fig. 1.**
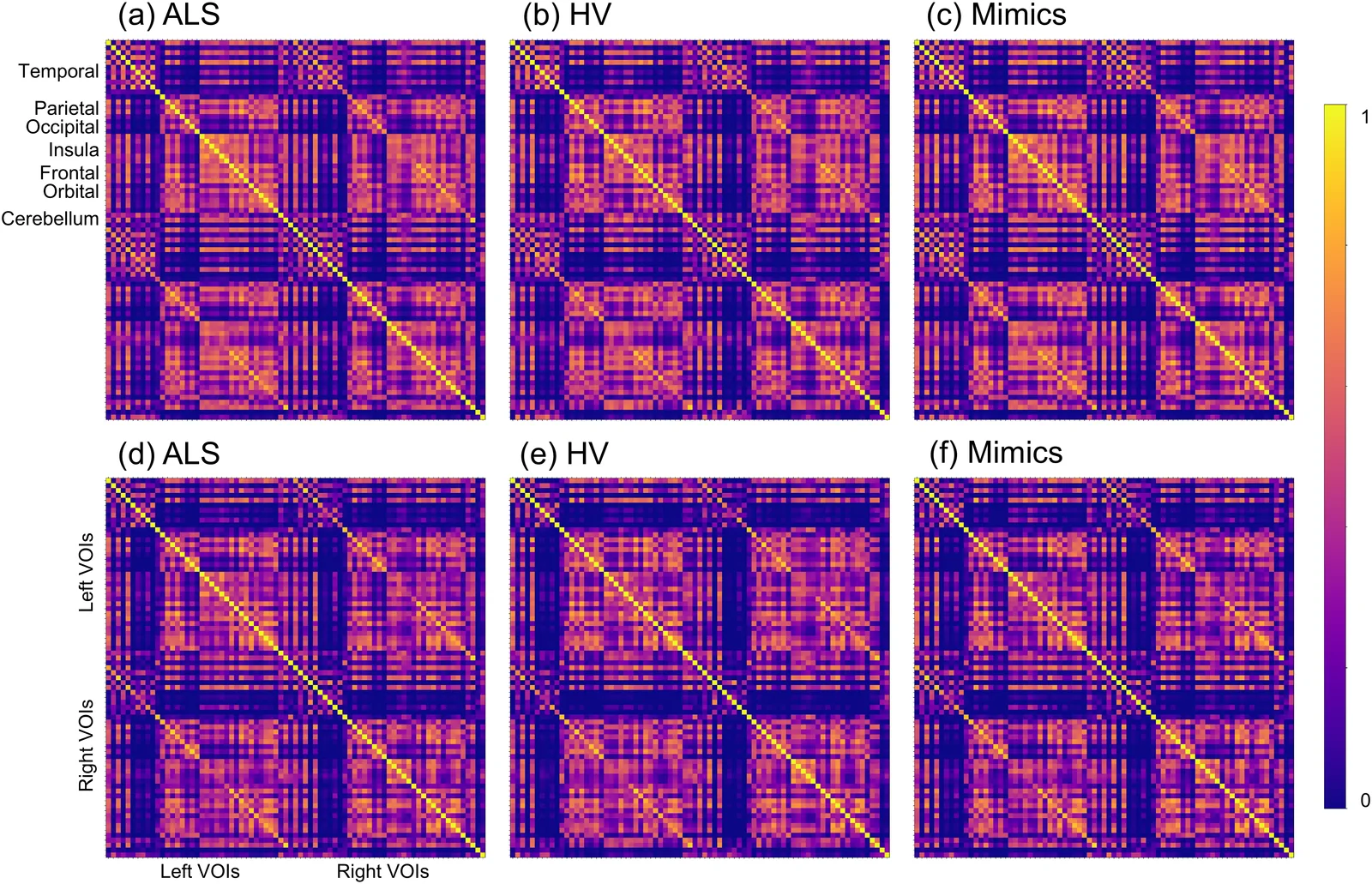
Average metabolic connectivity matrices based on Wasserstein distance (WD) for (a) ALS, (b) healthy volunteers (HV), and (c) ALS mimics, and based on KLSE for (d) ALS, (e) HV, and (f) ALS mimics.

**Fig. 2.**
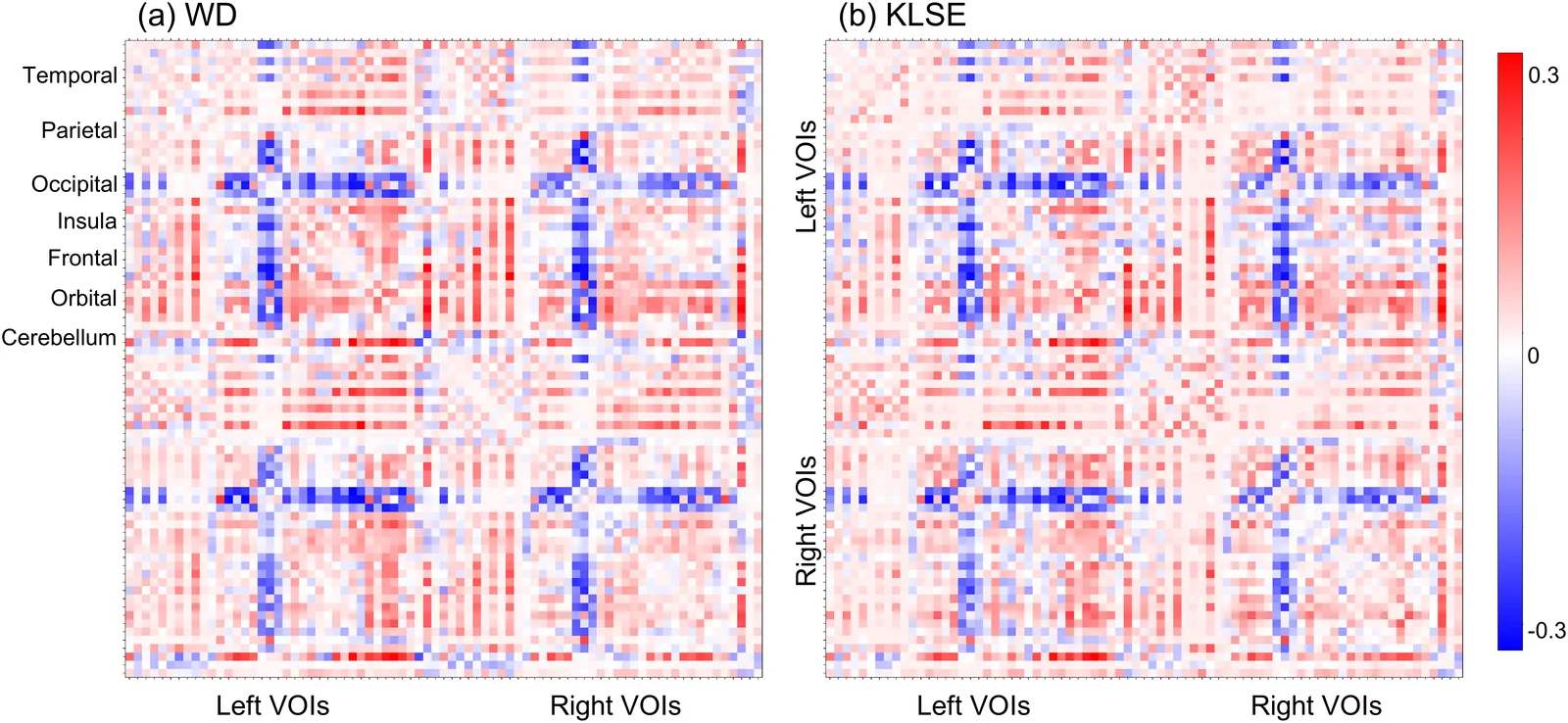
Standardised mean differences (Cohen’s *d*) between the healthy volunteer (HV) and age-matched ALS (ALS_amHV_) groups for (a) Wasserstein distance (WD)-based and (b) KLSE-based connectivity matrices.

Figure 1a–c shows the average WD-based connectivity matrices for the ALS, HV, and ALS mimics groups. Overall, the group-averaged matrices appeared broadly similar. However, closer inspection revealed that, relative to the HV group, the group-averaged matrix for the ALS group exhibited reduced connectivity between the occipital regions and frontal, parietal, and insular regions, together with an increased connectivity between frontal regions and temporal and insular regions. The cerebellum also demonstrated higher connectivity with most other regions in the ALS group than in the HV group. Maps of standardised mean differences (Cohen’s *d*) between the HV and age-matched ALS_amHV_ groups are shown in Figure 2a. By comparison, the group-averaged adjacency matrices for the ALS and ALS mimics groups showed no clear systematic increase or decrease in connectivity. Similar patterns were observed for the KLSE-based connectivity matrices across these groups (Figs. 1d–f and 2b).

Group differences were further assessed using an SVM-based classification with 10 repetitions of 10-fold cross-validation using randomly reshuffled datasets. For ALS_amHV_ versus HV, a classification using KLSE-based connectivity matrices achieved an overall accuracy of 0.82 ± 0.02 (mean ± SD), while an accuracy of 0.84 ± 0.01 was reached using WD-based matrices. Both accuracies were comparable with the classification performance obtained with voxel-wise PET data (0.86 ± 0.01), although a voxel-wise classification could still achieve a significantly higher performance than an approach using either WD- or KLSE-based matrices (p<0.05 ). For ALS versus ALS mimics, an SVM-based classification using KLSE-based matrices yielded an accuracy of 0.61 ± 0.02, while an approach using WD-based matrices achieved significantly higher accuracy of 0.65 ± 0.02 (p<0.001 ), with both methods significantly outperforming an image-based classification (p<0.01 ). An overview of classification performance metrics is given in Table 2.

**Table 2.** Results of SVM classification across 10 repetitions of 10-fold cross-validation for WD-based and KLSE-based connectivity matrices, alongside voxel-wise PET data.

	Sensitivity	Specificity	Overall accuracy	ROC-AUC
WD-based			
*ALS_amHV_ vs HV*	0.85 ± 0.02	0.82 ± 0.02	0.84 ± 0.01	0.92 ± 0.01
KLSE-based	0.83 ± 0.01	0.81 ± 0.03	0.82 ± 0.02	0.91 ± 0.01
voxel-wise PET	0.87 ± 0.01	0.85 ± 0.03	0.86 ± 0.01	0.93 ± 0.01
WD-based			
*ALS vs ALS mimics*	0.65 ± 0.02	0.67 ± 0.04	0.65 ± 0.02	0.68 ± 0.02
KLSE-based	0.62 ± 0.02	0.59 ± 0.05	0.61 ± 0.02	0.58 ± 0.01
voxel-wise PET	0.54 ± 0.02	0.50 ± 0.07	0.53 ± 0.02	0.55 ± 0.02

For both age-matched ALS (ALS_amHV_) vs healthy volunteers (HV) and ALS vs ALS mimics classifications, performance metrics differed significantly across the three methods (p<0.05), except for the specificity of ALS vs HV classification using WD- and KLSE-based connectivity matrices.

### 3.3 Correlations between WD-based and KLSE-based adjacency matrix and nodal metrics

**Fig. 3.**
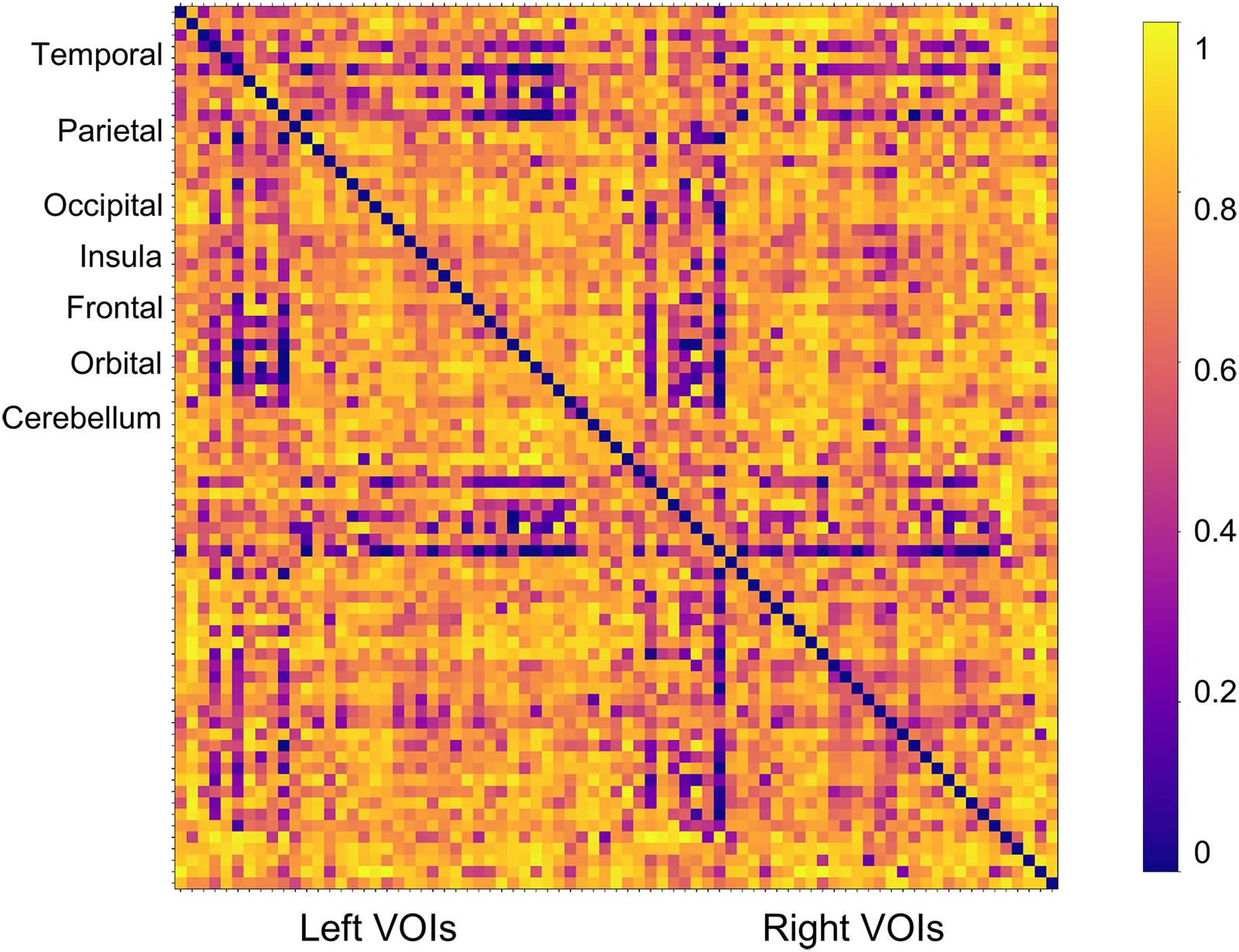
Edge-wise Pearson correlation coefficients between Wasserstein distance (WD)- and KLSE-based connectivity adjacency matrices across healthy volunteers. Each matrix element represents the correlation across subjects between the corresponding edges in the WD- and KLSE-based matrices.

Pearson’s *r* was computed for each corresponding edge between the paired KLSE- and WD-based adjacency matrices across subjects in the healthy volunteer group. The resulting edge-wise correlation matrix is shown in Figure 3. Overall, correlations exceeded 0.6 for the majority of edges, with 94% of the edges showing statistically significant correlations (p<0.05 , Supplementary Fig. S1), indicating substantial agreement between the two methods at the level of individual adjacency matrix entries.

The nodal metrics derived from the WD-based metabolic networks in the HV group showed strong correlations with the corresponding metrics obtained from the KLSE-based networks across all metrics examined (node strength, average path length, clustering coefficient, local efficiency, and betweenness centrality), with all correlations reaching high statistical significance (p<0.001 ). Table 3 summarises the Pearson’s correlation coefficient *r*.

**Table 3.** Pearson’s correlation coefficient *r* between nodal metrics derived from Wasserstein distance (WD)-based and KLSE-based metabolic networks in healthy volunteers.

	Pearson’s *r*
Node strength	0.86
Average path length	0.78
Clustering coefficient	0.86
Local efficiency	0.86
Betweenness centrality	0.36

For all comparisons, p<0.001.

### 3.4 Metabolic network nodal metrics in ALS_amHV_ versus healthy volunteers and ALS versus ALS mimics

**Fig. 4.**
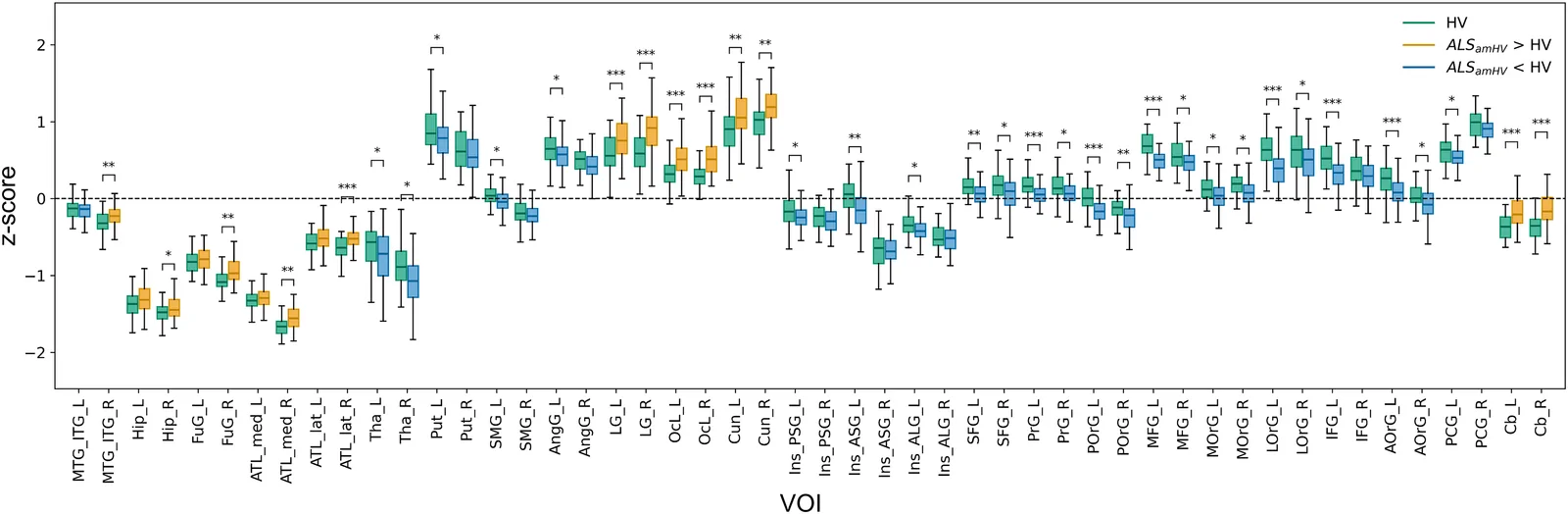
Average regional z-scores for the age-matched ALS (ALS_amHV_) group compared with the HV group. ALS_amHV_ > HV and ALS_amHV_ < HV indicate regions with significantly higher or lower z-scores in ALS_amHV_ compared with healthy volunteers, respectively. Significance levels are indicated as follows: ^*p<0.05 ^, ^**p<0.01 ^, ^***p<0.001 ^.

Compared with the HV group, the ALS_amHV_ group exhibited significantly higher regional z-scores of ^18^F-FDG uptake in the anterior temporal lobe, fusiform gyrus, hippocampus, and cerebellum, with shifts towards the global mean (*z* = 0), as well as in the occipital lobe, where values shifted further beyond the global mean (Fig. 4). Lower z-scores were observed in nearly all remaining regions, with the frontal and orbital frontal regions showing shifts towards the global mean, whereas the insula and thalamus demonstrated lower z-scores moving further away from the global mean. All reported regional differences remained significant after FDR correction for multiple comparisons.

**Fig. 5.**
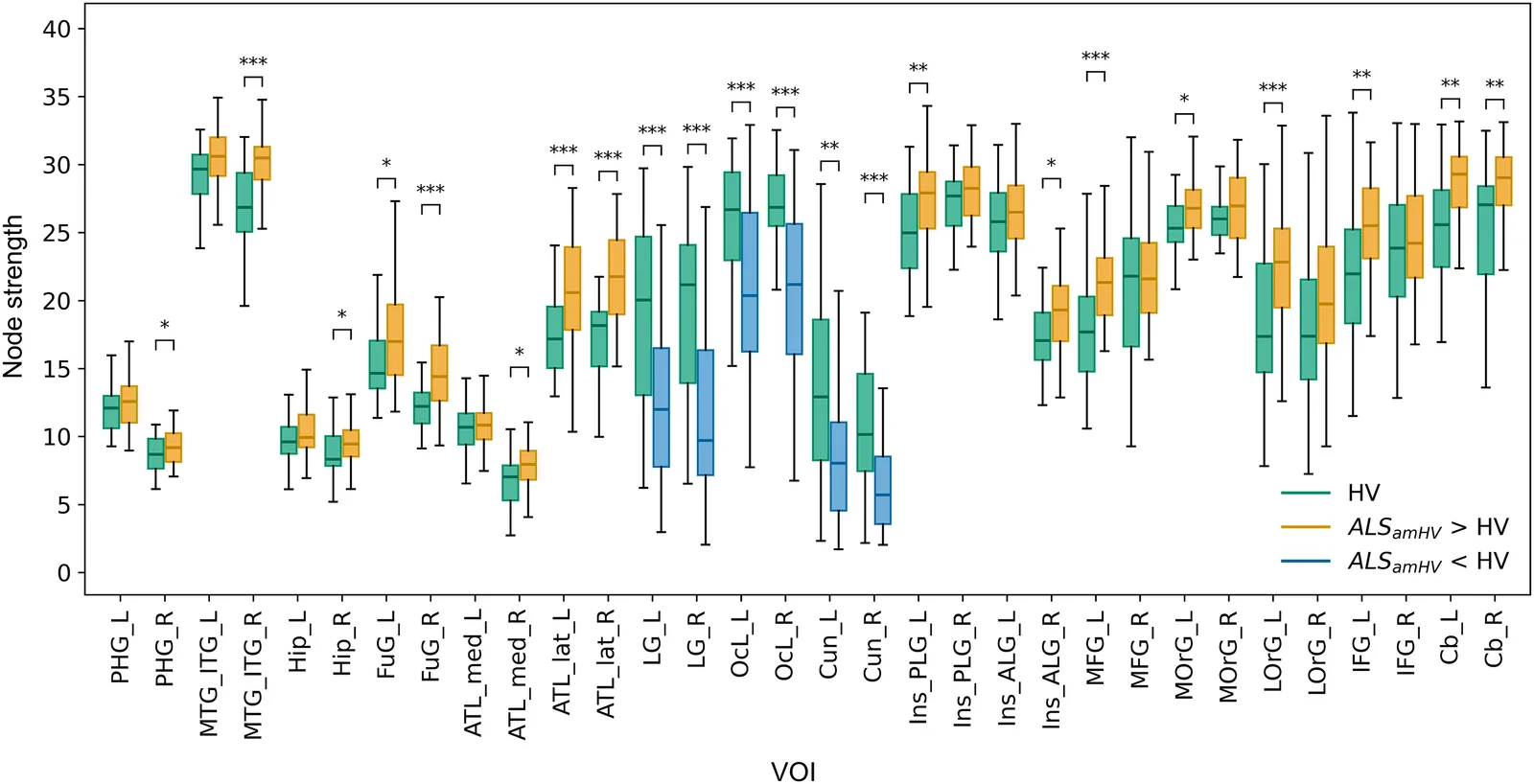
Node strength of the Wasserstein distance (WD)-based connectivity network for the age-matched ALS (ALS_amHV_) group compared with the HV group. Only regions showing significant group differences in at least one hemisphere are displayed. ALS_amHV_ > HV and ALS_amHV_ < HV indicate regions with significantly higher or lower node strength in ALS_amHV_ than in healthy volunteers, respectively. Significance levels are indicated as follows: *p<0.05 , **p<0.01 , ***p<0.001 .

Building on these metabolic findings, the ALS_amHV_ group also showed a significantly higher node strength in 16 regions based on the WD-based metabolic networks compared with the HV group (Fig. 5). The most pronounced increases in node strength were found in the temporal lobe, particularly in the right anterior, middle, and inferior temporal gyri, fusiform gyrus, hippocampus, and parahippocampal gyrus. Additional increases were observed in the middle and inferior frontal gyri and the medial and lateral orbital gyri, predominantly in the left hemisphere, as well as bilaterally in the insula and cerebellum. In contrast, the ALS_amHV_ group exhibited a significantly lower node strength in six regions of the occipital lobe. A comprehensive comparison of all regions is provided in Table 4. All differences remained significant after FDR correction for multiple comparisons.

**Table 4.** Node strengths in the Wasserstein distance (WD)-based metabolic connectivity networks of age-matched ALS (ALS_amHV_) patients compared with healthy volunteers (HV), reported as mean ± SD for each region in the left and right hemispheres, along with the corresponding asymmetry index (AI).

	ALS			HV		
	Left	Right	AI	Left	Right	AI
Superior temporal gyrus middle part	28.1 ± 3.1	29.4 ± 2.8	0 ± 0.1	27.6 ± 3.1	29.2 ± 2.0	0 ± 0.1
Superior temporal gyrus anterior part	13.5 ± 3.2	13.8 ± 3.3	0 ± 0.1	13.0 ± 2.3	12.7 ± 3.5	0 ± 0.1
Posterior temporal lobe	26.8 ± 2.4	25.6 ± 2.0	0 ± 0	26.7 ± 2.1	24.9 ± 2.4	0 ± 0.1
Parahippocampal and ambient gyrus	12.8 ± 2.5	9.5 ± 1.7^*^	-0.2 ± 0.1	12.0 ± 1.7	8.7 ± 1.4	-0.2 ± 0.1
Middle and inferior temporal gyrus	30.2 ± 3.2	30.1 ± 2.1^***^	0 ± 0.1	29.3 ± 2.2	26.6 ± 3.5	-0.1 ± 0.1
Hippocampus	10.5 ± 2.0	9.7 ± 1.8^*^	0 ± 0.1	9.8 ± 1.5	8.7 ± 1.7	-0.1 ± 0.1
Fusiform gyrus	17.3 ± 3.6^*^	14.8 ± 3.1^***^	-0.1 ± 0.1	15.4 ± 2.7	12.3 ± 2.0	-0.1 ± 0.1
Anterior temporal lobe medial part	10.9 ± 1.5	7.9 ± 1.6^*^	-0.2 ± 0.1	10.7 ± 1.6	6.8 ± 1.9	-0.2 ± 0.2
Anterior temporal lobe lateral part	20.7 ± 4.3^***^	21.7 ± 3.2^***^	0 ± 0.1	17.4 ± 2.9	17.2 ± 2.8	0 ± 0.1
Amygdala	9.2 ± 1.7	6.9 ± 1.5	-0.2 ± 0.1	8.7 ± 1.5	6.7 ± 1.5	-0.1 ± 0.1
Thalamus	12.1 ± 6.0	8.8 ± 4.6	-0.2 ± 0.1	13.5 ± 5.9	10.3 ± 5.1	-0.2 ± 0.1
Putamen	14.4 ± 5.8	19.3 ± 6.4	0.2 ± 0.1	12.8 ± 6.2	18.5 ± 6.3	0.2 ± 0.2
Supramarginal gyrus	30.1 ± 3.1	29.0 ± 2.3	0 ± 0.1	29.6 ± 1.9	27.9 ± 2.7	0 ± 0.1
Superior parietal gyrus	25.3 ± 4.9	26.1 ± 4.7	0 ± 0	24.7 ± 4.1	24.9 ± 4.2	0 ± 0
Postcentral gyrus	30.2 ± 3.1	29.1 ± 3.2	0 ± 0	30 ± 1.8	28.9 ± 2.1	0 ± 0
Angular gyrus	19.2 ± 4.9	22.1 ± 5.2	0.1 ± 0.1	18.2 ± 5.4	21.9 ± 4.2	0.1 ± 0.2
Lingual gyrus	12.5 ± 6.0^***^	11.4 ± 6.2^***^	-0.1 ± 0.1	19.1 ± 6.6	19.1 ± 6.8	0 ± 0.1
Lateral remainder occipital lobe	20.8 ± 6.4^***^	20.5 ± 6.6^***^	0 ± 0.1	26.2 ± 4.0	26.6 ± 3.7	0 ± 0.1
Cuneus	8.8 ± 5.1^**^	6.4 ± 3.5^***^	-0.1 ± 0.3	13.4 ± 6.3	10.9 ± 5.4	-0.1 ± 0.2
Insula posterior short gyrus	28.5 ± 2.7	25.4 ± 2.7	-0.1 ± 0.1	27.7 ± 3.0	24.4 ± 2.7	-0.1 ± 0.1
Insula posterior long gyrus	27.4 ± 3.2^**^	27.8 ± 3.0	0 ± 0.1	25.1 ± 3.2	26.8 ± 3.5	0 ± 0.1
Insula middle short gyrus	27.2 ± 2.2	24.8 ± 3.5	-0.1 ± 0.1	26.7 ± 2.3	23.6 ± 3.3	-0.1 ± 0.1
Insula anterior short gyrus	28.2 ± 3.7	19.3 ± 4.6	-0.2 ± 0.1	27.9 ± 2.8	17.6 ± 4.0	-0.2 ± 0.1
Insula anterior long gyrus	26.5 ± 3.1	19.2 ± 3.1^*^	-0.2 ± 0.1	25.6 ± 2.8	17.3 ± 3.2	-0.2 ± 0.1
Insula anterior inferior cortex	23.6 ± 2.8	25.5 ± 2.6	0 ± 0.1	23.8 ± 3.5	25.1 ± 2.7	0 ± 0.1
Superior frontal gyrus	29.7 ± 2.4	28.7 ± 2.8	0 ± 0	28.6 ± 2.3	28.2 ± 2.6	0 ± 0
Straight gyrus	27.2 ± 2.5	28.7 ± 2.3	0 ± 0	27.2 ± 2.1	28.0 ± 1.6	0 ± 0
Precentral gyrus	30.1 ± 2.7	28.8 ± 3.0	0 ± 0	29.1 ± 2.1	28.3 ± 2.7	0 ± 0.1
Posterior orbital gyrus	29.2 ± 2.5	28.8 ± 2.7	0 ± 0.1	29.1 ± 1.9	28.8 ± 2.2	0 ± 0
Middle frontal gyrus	21.6 ± 3.8^***^	22.3 ± 4.4	0 ± 0.1	17.7 ± 4.2	20.8 ± 5.3	0.1 ± 0.2
Medial orbital gyrus	26.8 ± 2.2^*^	26.9 ± 2.5	0 ± 0	25.4 ± 2.1	25.8 ± 2.1	0 ± 0
Lateral orbital gyrus	22.8 ± 4.3^***^	20.8 ± 5.6	-0.1 ± 0.1	18.2 ± 5.2	18.1 ± 5.8	0 ± 0.2
Inferior frontal gyrus	25.5 ± 3.5^**^	24.8 ± 4.0	0 ± 0.1	21.8 ± 5.1	23.4 ± 5.1	0 ± 0.2
Anterior orbital gyrus	27.4 ± 3.1	24.9 ± 3.2	-0.1 ± 0.1	26.5 ± 4.0	25.6 ± 3.8	0 ± 0.1
Posterior cingulate gyrus	19.4 ± 3.9	11.6 ± 4.0	-0.3 ± 0.1	19.0 ± 5.0	11.4 ± 5.5	-0.3 ± 0.2
Anterior cingulate gyrus	20.2 ± 4.2	25.7 ± 5.0	0.1 ± 0.1	20.5 ± 4.8	25.5 ± 4.7	0.1 ± 0.1
Cerebellum	28.4 ± 3.4^**^	28.2 ± 3.6^**^	0 ± 0	25.4 ± 4.1	25.3 ± 4.5	0 ± 0
Caudate nucleus	8.4 ± 5.2	3.3 ± 2.9	-0.5 ± 0.2	9.8 ± 5.5	4.9 ± 3.9	-0.4 ± 0.2
Brainstem excluding substantia nigra^†^	13.3 ± 3.3	/	12.5 ± 2.2	/		

Significant group difference ^*^p<0.05; ^**^p<0.01; ^***^p<0.001.

† The brainstem was considered as a whole region and not analysed as a left–right pair.

**Fig. 6.**
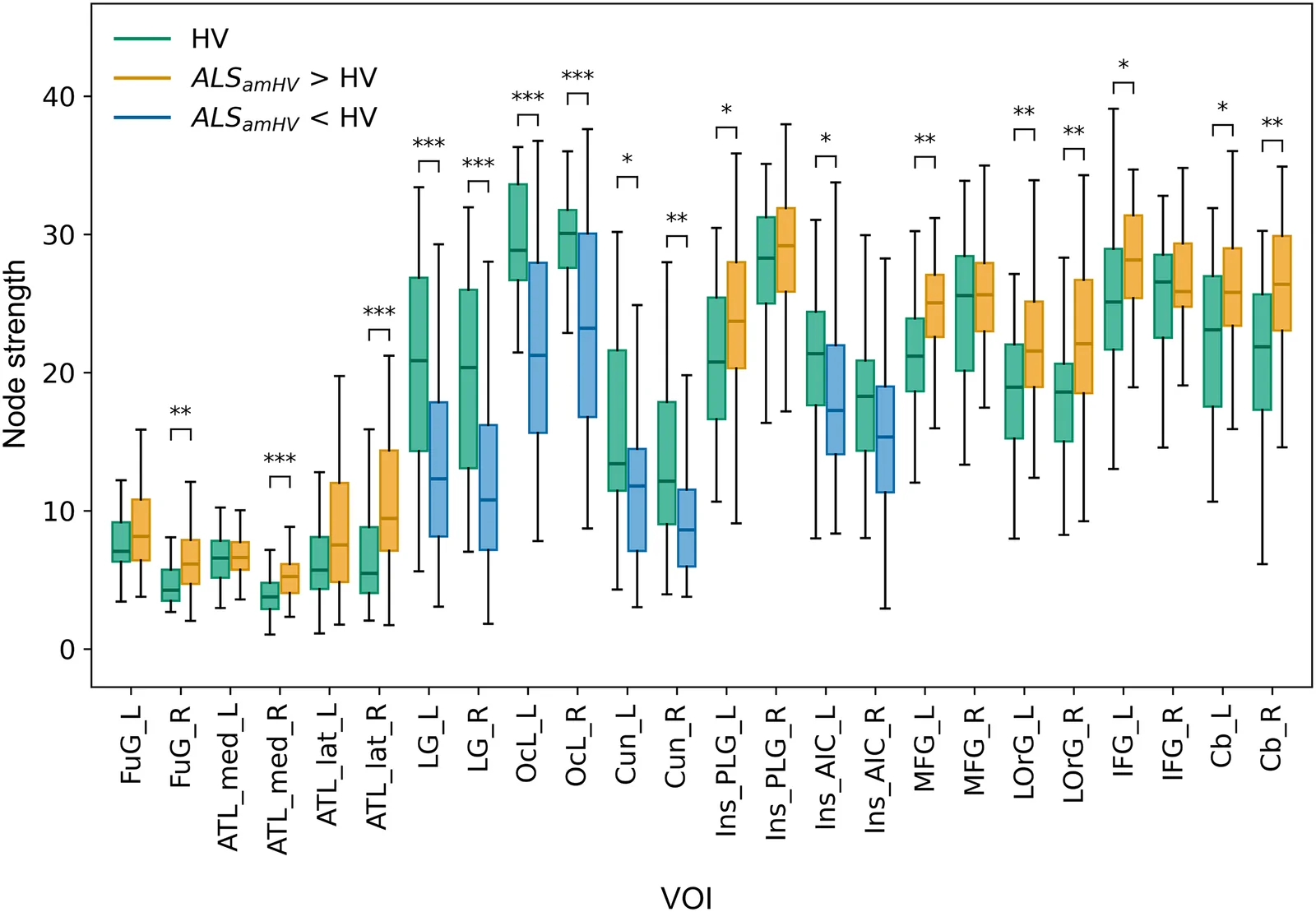
Node strength of the KLSE-based connectivity network for the age-matched ALS (ALS_amHV_) group compared with the HV group. Only regions showing significant group differences in at least one hemisphere are displayed. ALS_amHV_ > HV and ALS_amHV_ < HV indicate regions with significantly higher or lower node strength in ALS_amHV_ than in healthy volunteers, respectively. Significance levels are indicated as follows: ^*p<0.05 ^, ^**p<0.01 ^, ^***p<0.001 ^.

Consistent with the WD-based results, similar patterns were observed in the KLSE-based metabolic networks. The ALS_amHV_ group demonstrated a significantly higher node strength in 10 regions, including the anterior temporal lobe and fusiform gyrus in the right hemisphere, the middle short gyrus of the left insula, frontal and orbital gyri predominantly in the left hemisphere, and the cerebellum bilaterally. Significantly lower values were found in seven regions, again in the occipital lobe of both hemispheres and in the left anterior inferior insular cortex (Fig. 6). All reported differences remained significant after FDR correction for multiple comparisons.

Similar patterns were observed for the clustering coefficient (Supplementary Figs. S2 and S3) and local efficiency (Supplementary Figs. S4 and S5), which showed regional alterations consistent with their positive correlations with node strength. In contrast, average path length showed inverse patterns (Supplementary Figs. S6 and S7), consistent with its negative correlation with node strength. Betweenness centrality was the least sensitive metric to network alterations, with the ALS_amHV_ group showing significantly increased betweenness centrality only for the WD-based networks in the left superior parietal gyrus and the right lateral orbital gyrus compared with the HV group.

Across all five analysed nodal metrics, no significant differences were identified between the ALS and ALS mimics groups for either the WD-based or KLSE-based metabolic connectivity networks after FDR correction for multiple comparisons.

## 4 Discussion

The aims of this study were threefold. First, we sought to introduce the Wasserstein distance (WD) as a practical alternative to KLSE for constructing subject-specific metabolic connectivity networks from static brain ^18^F-FDG PET scans, and to demonstrate that WD provides at least equivalent information while being easier to implement. Second, we aimed to compare WD-based and KLSE-based metabolic connectivity matrices in two clinically relevant classification tasks, namely differentiation between ALS_amHV_ and healthy volunteers (HV) and between ALS and ALS mimics, in order to assess the extent to which both approaches preserve disease-relevant information. Third, we evaluated whether graph theoretical metrics derived from these metabolic connectivity matrices could improve classification performance and, importantly, provide meaningful insights into disease-specific uptake patterns based on static brain ^18^F-FDG PET scans.

To address these aims, WD was used to quantify distances between regional distributions of FDG uptake, which enabled the construction of metabolic networks in which connectivity strength is inversely proportional to these distances. This approach allowed individual metabolic connectivity networks to be derived for each subject while offering several advantages over KLSE-based methods. As outlined in the Introduction, a key advantage of the Wasserstein distance over KLSE is its ability to robustly compare regional uptake distributions even for the non-overlap parts. Whereas KLSE relies on probability density function estimation and is primarily sensitive to overlapping distributional components, often yielding infinite divergence or zero similarity for non-overlapping parts in the two distributions, Wasserstein distance remains well defined and captures distributional differences across the full support. Moreover, it can be computed directly from voxel-wise uptake values without requiring probability density function estimation and histogram binning.

The connectivity matrices were subsequently used to construct undirected weighted networks for each subject, which were then assessed with nodal metrics from graph theory. The strong correlations observed between nodal metrics derived from metabolic connectivity networks constructed using WD and KLSE in the HV group indicate a high degree of concordance between the two approaches. This consistency suggests that both frameworks capture largely comparable topological features at the nodal level, thereby providing confidence that the observed patterns are not driven by method-specific artefacts but instead reflect shared structural features of the data.

However, it remains important to note that molecular connectivity measures based on distribution similarity differ conceptually from traditional connectivity measures derived from resting-state fMRI time-series correlations or from group-level covariance analyses. The rationale for interpreting these distribution-similarity–based measures as molecular connectivity lies in the assumption that regions exhibiting similar tracer uptake distributions may share common underlying biological substrates, such as comparable receptor density, metabolic demand, or vulnerability to disease-related processes. Distribution-similarity–based connectivity, therefore, captures a complementary aspect of brain organisation, reflecting the degree to which molecular processes are distributed coherently across regions within an individual. While these measures should not be interpreted as direct analogues of temporal or functional connectivity, they provide a compact and interpretable representation of molecular heterogeneity, particularly in settings where dynamic or longitudinal information is not available.

At the same time, the extent to which graph-derived features reflect network-level organisation beyond regional metabolic shifts remains an important consideration. In this regard, the comparison between voxel-wise PET-based classification and connectivity-based classification is informative. While both approaches yielded comparable performance for ALS_amHV_ versus HV, the connectivity-based representations showed better classification performance in the more challenging ALS versus ALS mimics comparison. For this more challenging classification task, both connectivity-based approaches achieved accuracies above 0.60, whereas voxel-wise image-based classification performed at approximately chance level. This suggests that the graph-based representation is not simply a restatement of local uptake differences, but may preserve informative inter-regional structure in a compact form, even though part of the signal captured by the nodal metrics remains rooted in underlying regional metabolic changes. At the same time, these ALS versus mimics findings should be interpreted as proof-of-concept in a difficult and heterogeneous differential-diagnostic setting rather than as evidence of diagnostic readiness, and additional validation in larger and prospectively characterised cohorts will be required to determine whether this approach can achieve sufficient robustness and performance for clinical use. Formal comparisons between regional-only, graph-based, and combined models would also be valuable in future work to further disentangle regional and network-level contributions.

This interpretation is consistent with the regional pattern of nodal alterations observed in the ALS_amHV_ group. Compared with the HV group, the ALS_amHV_ group exhibited significantly higher nodal strength values in the temporal lobe, middle and inferior frontal gyri, medial and lateral orbital gyri, and the cerebellum, together with significantly lower values in the occipital lobe. These spatial patterns were consistent with the regional z-score differences between ALS_amHV_ and HV. More specifically, elevated z-scores in cerebellar and temporal regions, combined with reduced z-scores in frontal and orbital regions, shifted the regional intensity distributions towards the global mean (*z* = 0). As the distributions converged to the global mean, the WD and KL divergence relative to other regions decreased, which led to increased connectivity strength. In contrast, occipital regions showed elevated z-scores further away from the global mean, resulting in greater distributional separation from other regions, and consequently causing a corresponding reduction in connectivity strength. The close correspondence between regional nodal strength and regional z-scores supports a straightforward interpretation of these graph-theoretical metrics in terms of underlying FDG uptake patterns, with increases in connectivity strength reflecting convergence towards the global metabolic profile and decreases reflecting divergence from it.

Notably, significant differences were detected in more regions using the WD-based networks (22 regions) than using the KLSE-based networks (17 regions). Although this pattern may indicate that WD captures certain regional metabolic alterations somewhat more readily in the present dataset, these findings should be interpreted cautiously and do not by themselves establish superior disease sensitivity.

Despite the promising performance and interpretability of the proposed framework, this study also has several key limitations. First, the datasets were essentially acquired on a single PET/CT system. While this limits technical variability and enables a controlled comparison, it also constrains conclusions about generalisability. Multicentre validation across different PET/CT systems is, therefore, needed to evaluate robustness and sensitivity to differences in acquisition and reconstruction protocols. Second, other strategies for node representation and individual graph construction from static FDG-PET have been proposed (Tuan et al., 2026) but were not examined in this study. This was an intentional choice to keep the scope manageable and to allow a focused comparison between two distribution-based approaches that exploit the full within-VOI uptake profile, thereby capturing regional interactions beyond mean signal differences. Within this scope, KLSE served as a widely used reference in metabolic connectivity research, albeit requiring an explicit kernel density estimation step, whereas WD provides a principled alternative that quantifies distributional differences without an explicit kernel density estimation procedure. Finally, the classification analyses were intentionally restricted to imaging data alone, in order to evaluate the discriminatory value of voxel-wise PET features and connectivity-derived metrics in isolation. As a result, additional potential confounders or complementary predictors, such as sex, disease duration, medication use, ALSFRS-R, and other clinical variables, were not jointly modeled. Incorporating such factors would require a multivariable framework capable of combining high-dimensional imaging features with non-imaging covariates in a robust and interpretable manner. In addition, the ALS mimics cohort was diagnostically highly heterogeneous, comprising a broad spectrum of neurological, systemic, and functional disorders representative of real-world differential diagnosis. While this increases the clinical relevance of the comparison, it also likely contributed to the limited classification performance for ALS versus mimics, as these disorders differ substantially in their underlying biology and in the availability and interpretability of disease-specific clinical variables. Many potentially relevant clinical parameters were not uniformly available, not standardised across diagnoses, or not meaningfully comparable between ALS and non-ALS conditions. Prospective studies with harmonised acquisition protocols and standardised multimodal clinical phenotyping will, therefore, be needed to determine whether combining PET with non-imaging variables can further improve discrimination between ALS and ALS mimics.

Building on these findings, future work could extend the WD-based approach to other clinical cohorts to further characterise pathological alterations in metabolic brain networks across neurodegenerative diseases. Applying the method to additional PET tracers would make it possible to investigate molecular connectivity patterns associated with specific neurotransmitter systems, neuroreceptors, and proteinopathies. Moreover, future studies could broaden the methodological comparison by including additional graph-construction methods for individual metabolic network analysis, thereby further benchmarking how node definitions and similarity measures influence network topology and downstream clinical utility. In parallel, recent advances in PET imaging technology, providing substantially higher temporal and spatial resolution, open new avenues for network analysis using kinetic parametric imaging (Volpi et al., 2021, 2023) and for exploring functional molecular connectivity through functional dynamic PET scanning (Jamadar et al., 2019, 2021). Together, these developments provide a promising foundation for establishing WD-based molecular connectivity as a versatile framework for probing disease mechanisms, refining diagnostic markers, and potentially tracking disease progression across a range of neurological disorders. Finally, investigating relationships between network properties and clinical scores could further strengthen the reliability of the proposed framework, enhance interpretation of brain connectivity graphs derived from static FDG-PET scans, and clarify their potential role in clinical practice.

## Supplementary Material

Supplementary Material

## Data Availability

The datasets generated and analysed during the study are not publicly available due to GDPR, but are available from the corresponding authors on reasonable request.
