# Supplementary material for: Metabolic connectivity alterations in amyotrophic lateral sclerosis: Individual network analysis based on Wasserstein distances
Source: Imaging Neurosci (Camb). 2026 Aug 20;4:IMAG.a.1343. doi: 10.1162/IMAG.a.1343 (PMC13494962; doi:10.1162/IMAG.a.1343)
Supplement: Supplementary file 1 [file IMAG.a.1343_supp.pdf]

Supplementary Material

Table S1: Final diagnoses of ALS mimic cases.

**Final diagnosis of ALS Mimics patients**	**Number of subjects**
Spinal and Radicular Pathologies	7
Peripheral Neuropathies & Mononeuropathies	8
Psychiatric & Functional Neurological Disorders	4
Systemic Disease	2
Neuromuscular Disorder	1
Central Neurodegenerative and Movement Disorders	1
Other*	2
Total	25

* Other: transient drop foot; post-radiation tissue damage in neck muscles; orthopedic complication.

Table S2: Volumes (number of voxels) of atlas regions included in the analysis with a voxel corresponding to 8 mm^3.

	Number of Voxels	
	Left	Right
superior temporal gyrus middle part	1456	1368
superior temporal gyrus anterior part	600	623
posterior temporal lobe	5042	5081
parahippocampal and ambient gyrus	625	621
middle and inferior temporal gyrus	1878	1982
hippocampus	349	386
fusiform gyrus	610	611
anterior temporal lobe medial part	921	959
anterior temporal lobe lateral part	480	511
amygdala	241	219
thalamus	582	600
putamen	648	648
supramarginal gyrus	2570	2344
superior parietal gyrus	3810	4038
postcentral gyrus	2076	1893
angular gyrus	1634	1769
lingual gyrus	1468	1501
lateral remainder occipital lobe	4050	3973
cuneus	1059	1068
insula posterior short gyrus	205	223
insula posterior long gyrus	244	239
insula middle short gyrus	133	130
insula anterior short gyrus	238	220
insula anterior long gyrus	268	263
insula anterior inferior cortex	262	260
superior frontal gyrus	5384	5292
straight gyrus	356	390
precentral gyrus	2489	2372
posterior orbital gyrus	576	600
middle frontal gyrus	4465	4564
medial orbital gyrus	580	579
lateral orbital gyrus	391	452
inferior frontal gyrus	2081	1956
anterior orbital gyrus	681	686
posterior cingulate gyrus	913	895
anterior cingulate gyrus	945	932
cerebellum	8891	8787
caudate nucleus	600	624
brainstem excluding substantia nigra	249	

Table S3: Hyperparameters for SVM-based classifiers with 10-fold cross-validation using WD- and KLSE-based connectivity matrices, as well as voxel-wise PET data. Hyperparameters were selected on a small subset of the data prior to cross-validation.

	Class Weight	C
ALS_{amHV} vs HV		
WD-based	2.9	$4{\times}10^{-3}$
KLSE-based	16.5	$4{\times}10^{-3}$
voxel-wise PET	2.0	7.0
ALS vs ALS mimics		
WD-based	22.5	10^{-3}
KLSE-based	7.7	10^{-4}
voxel-wise PET	7.7	1.0

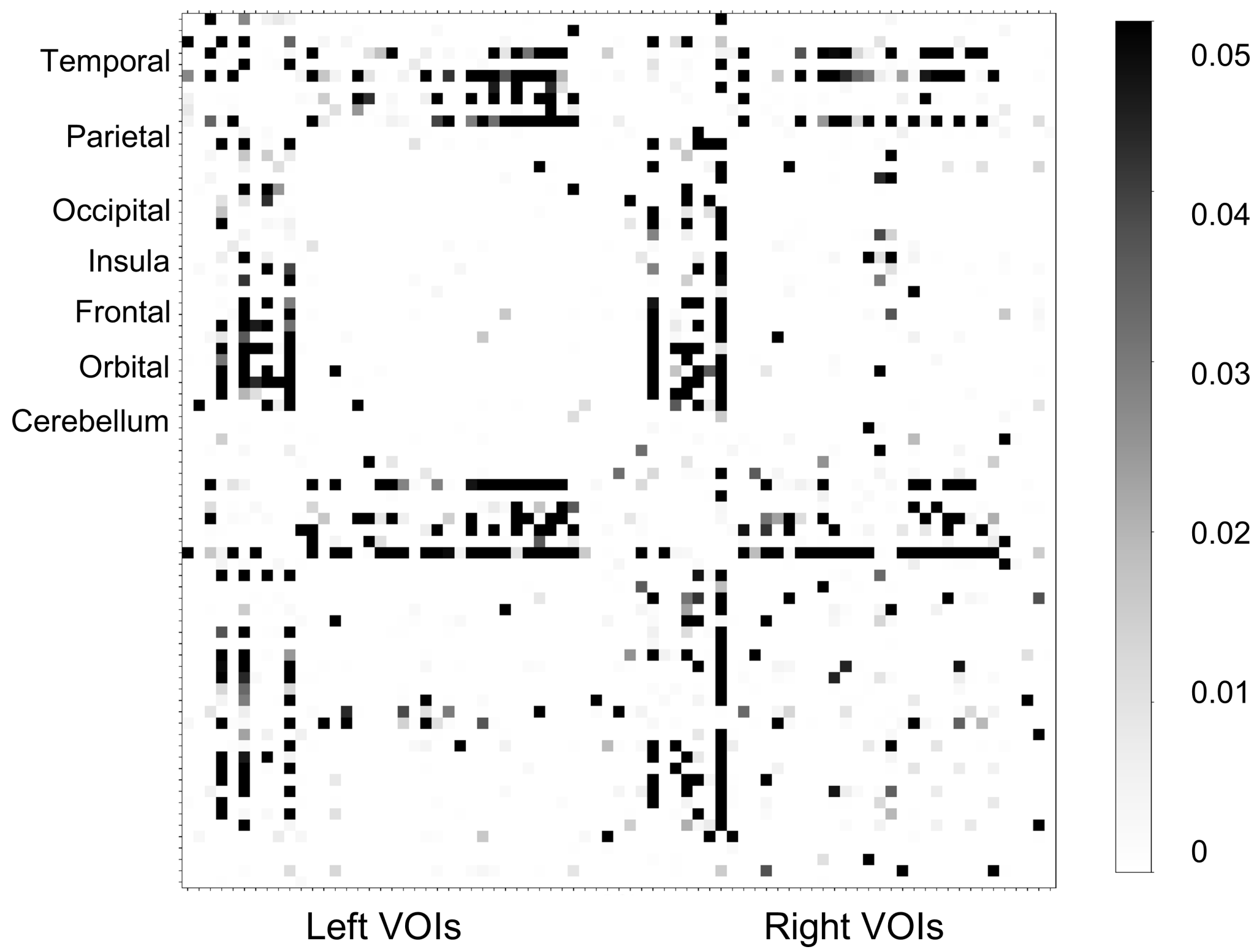

Figure S1: Edge-wise p-values corresponding to the Pearson correlation analysis between Wasserstein distance (WD)- and KLSE-based connectivity adjacency matrices across healthy volunteers. Each matrix element represents the statistical significance of the correlation across subjects between the corresponding edges in the WD- and KLSE-based matrices.

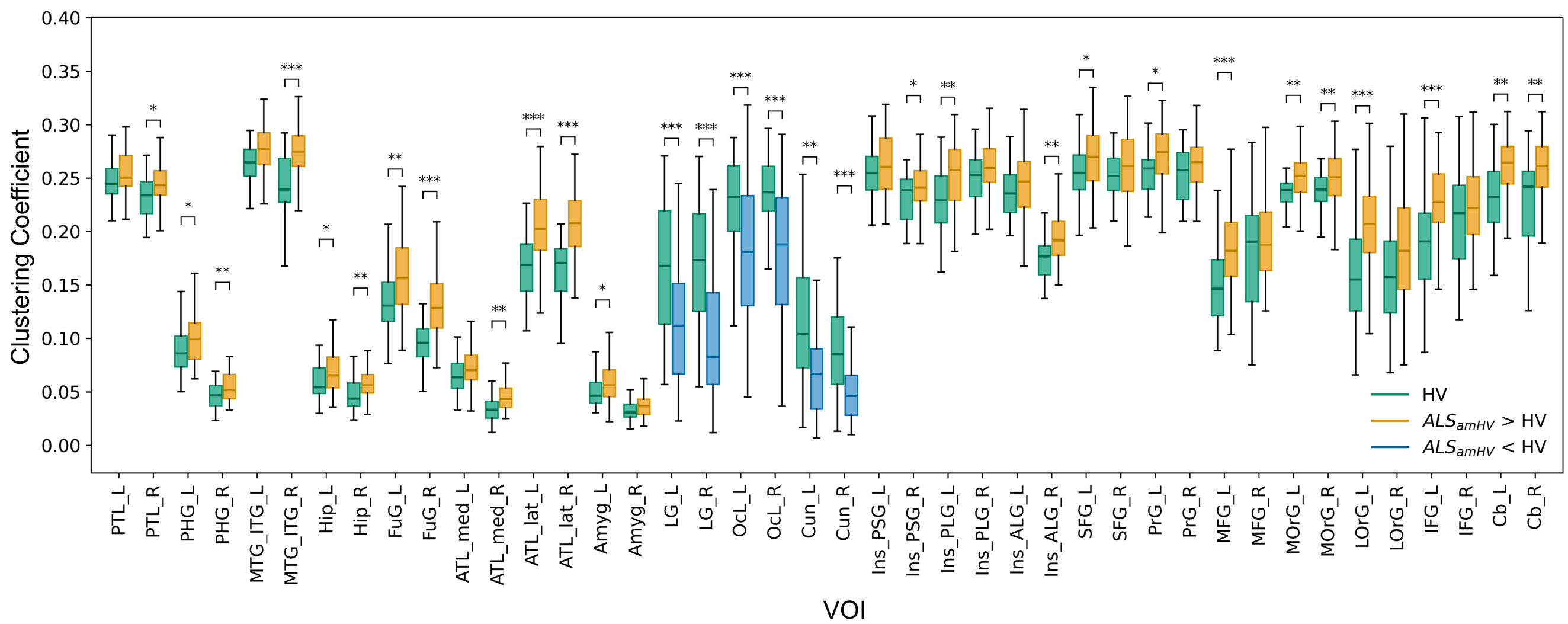

Figure S2: Nodal clustering coefficient of the Wasserstein distance (WD)–based connectivity network for the age-matched ALS ($\mathrm{ALS_{amHV}}$) group compared with the HV group. Only regions showing significant group differences in at least one hemisphere are displayed. $\mathrm{ALS_{amHV}} > \mathrm{HV}$ and $\mathrm{ALS_{amHV}} < \mathrm{HV}$ indicate regions with significantly higher or lower clustering coefficient in $\mathrm{ALS_{amHV}}$ compared with healthy volunteers, respectively. Significance levels are indicated as follows: $^{*}p < 0.05$, $^{**}p < 0.01$, $^{***}p < 0.001$

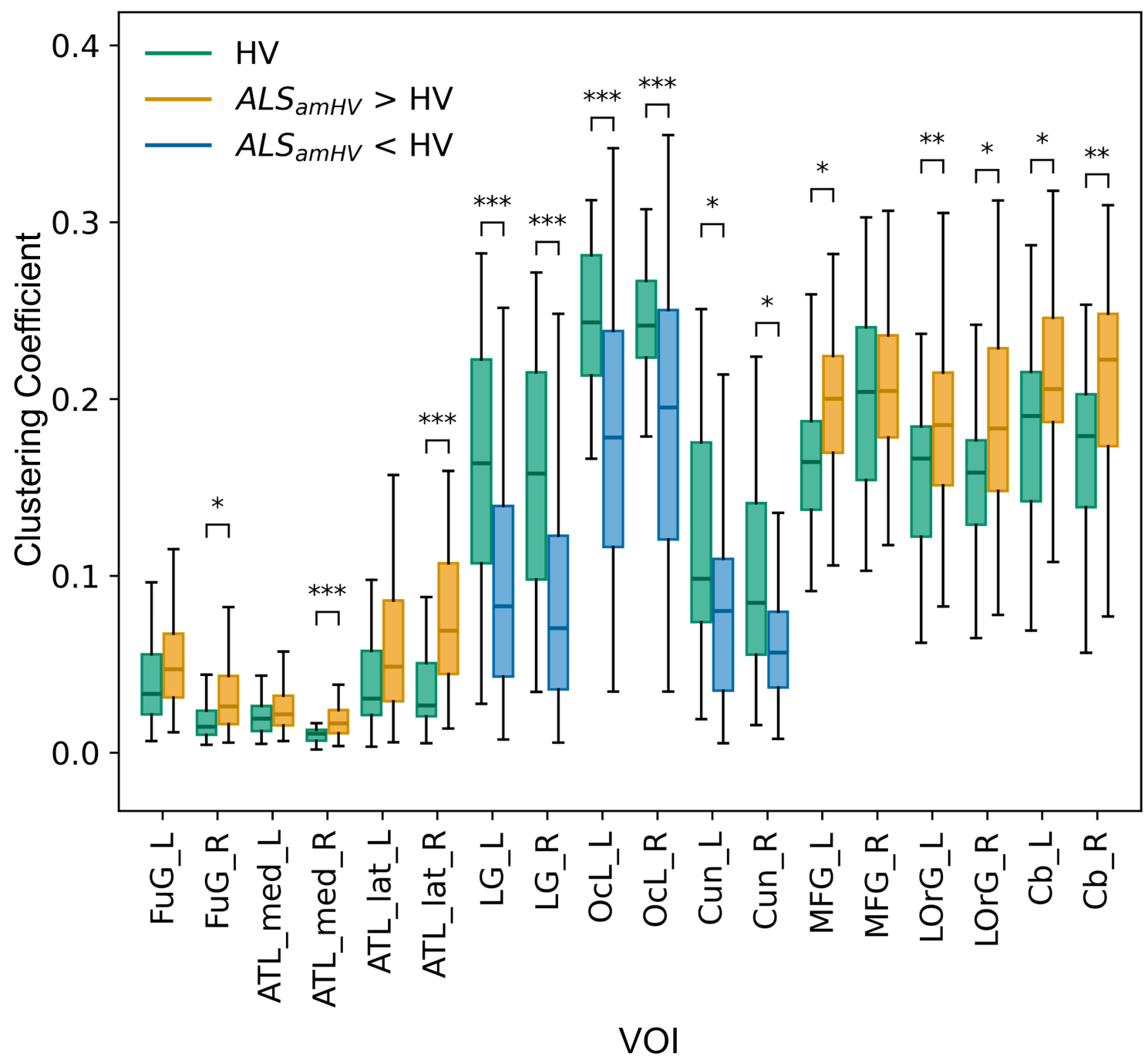

Figure S3: Nodal clustering coefficient of the KLSE–based connectivity network for the age-matched ALS (ALS_{amHV}) group compared with the HV group. Only regions showing significant group differences in at least one hemisphere are displayed. $ALS_{amHV} > HV$ and $ALS_{amHV} < HV$ indicate regions with significantly higher or lower clustering coefficient in ALS_{amHV} compared with healthy volunteers, respectively. Significance levels are indicated as follows: $^{*}p < 0.05$, $^{**}p < 0.01$, $^{***}p < 0.001$

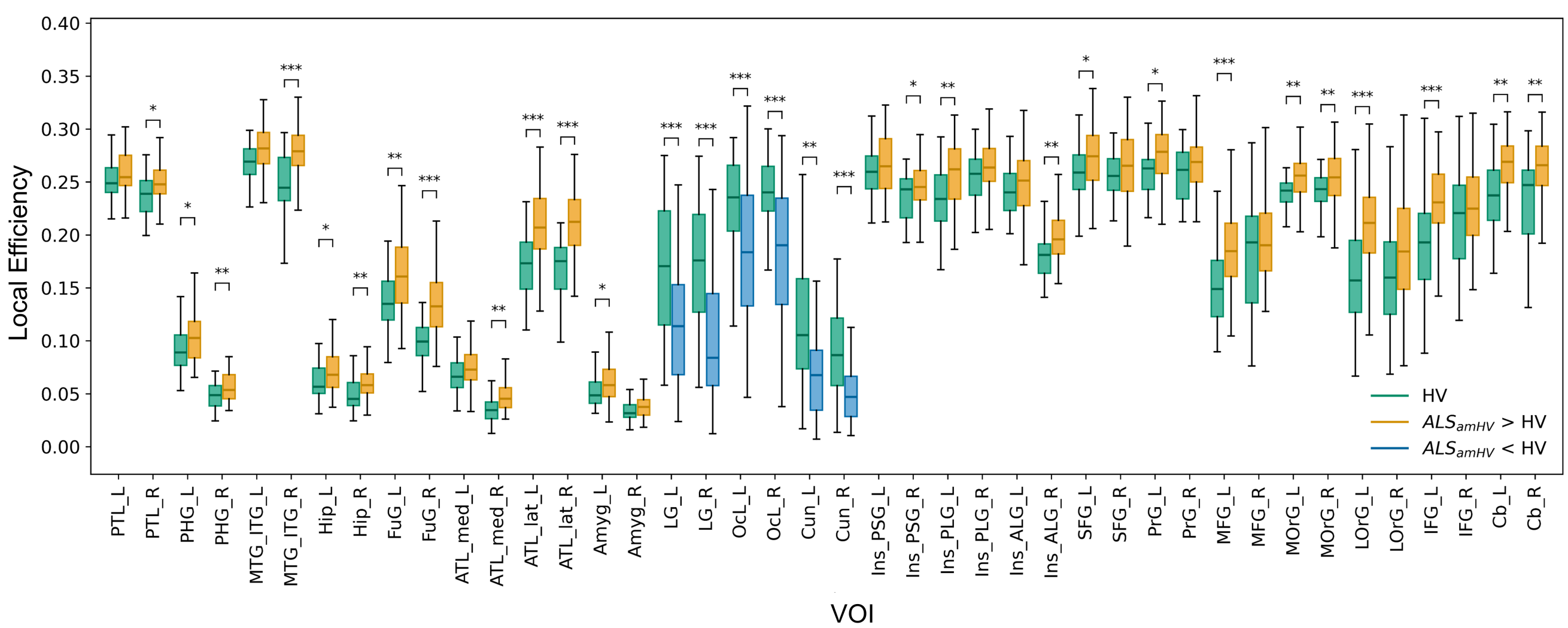

Figure S4: Nodal local efficiency of the Wasserstein distance (WD)–based connectivity network for the age-matched ALS (ALS_{amHV}) group compared with the HV group. Only regions showing significant group differences in at least one hemisphere are displayed. $ALS_{amHV} > HV$ and $ALS_{amHV} < HV$ indicate regions with significantly higher or lower local efficiency in ALS_{amHV} compared with healthy volunteers, respectively. Significance levels are indicated as follows: $^{*}p < 0.05$, $^{**}p < 0.01$, $^{***}p < 0.001$

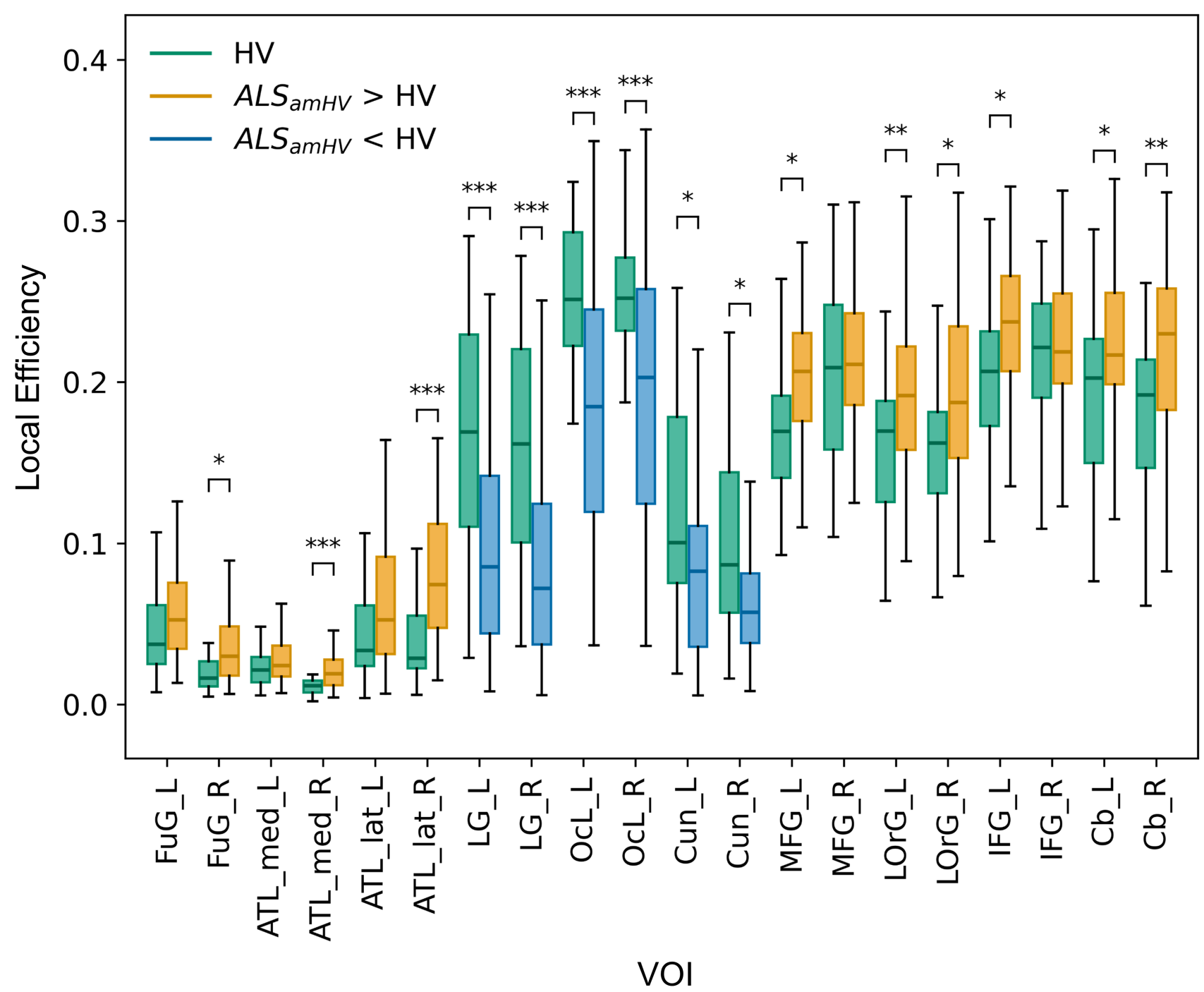

Figure S5: Nodal local efficiency of the KLSE–based connectivity network for the age-matched ALS (ALS_{amHV}) group compared with the HV group. Only regions showing significant group differences in at least one hemisphere are displayed. $ALS_{amHV} > HV$ and $ALS_{amHV} < HV$ indicate regions with significantly higher or lower local efficiency in ALS_{amHV} compared with healthy volunteers, respectively. Significance levels are indicated as follows: $^{*}p < 0.05$, $^{**}p < 0.01$, $^{***}p < 0.001$

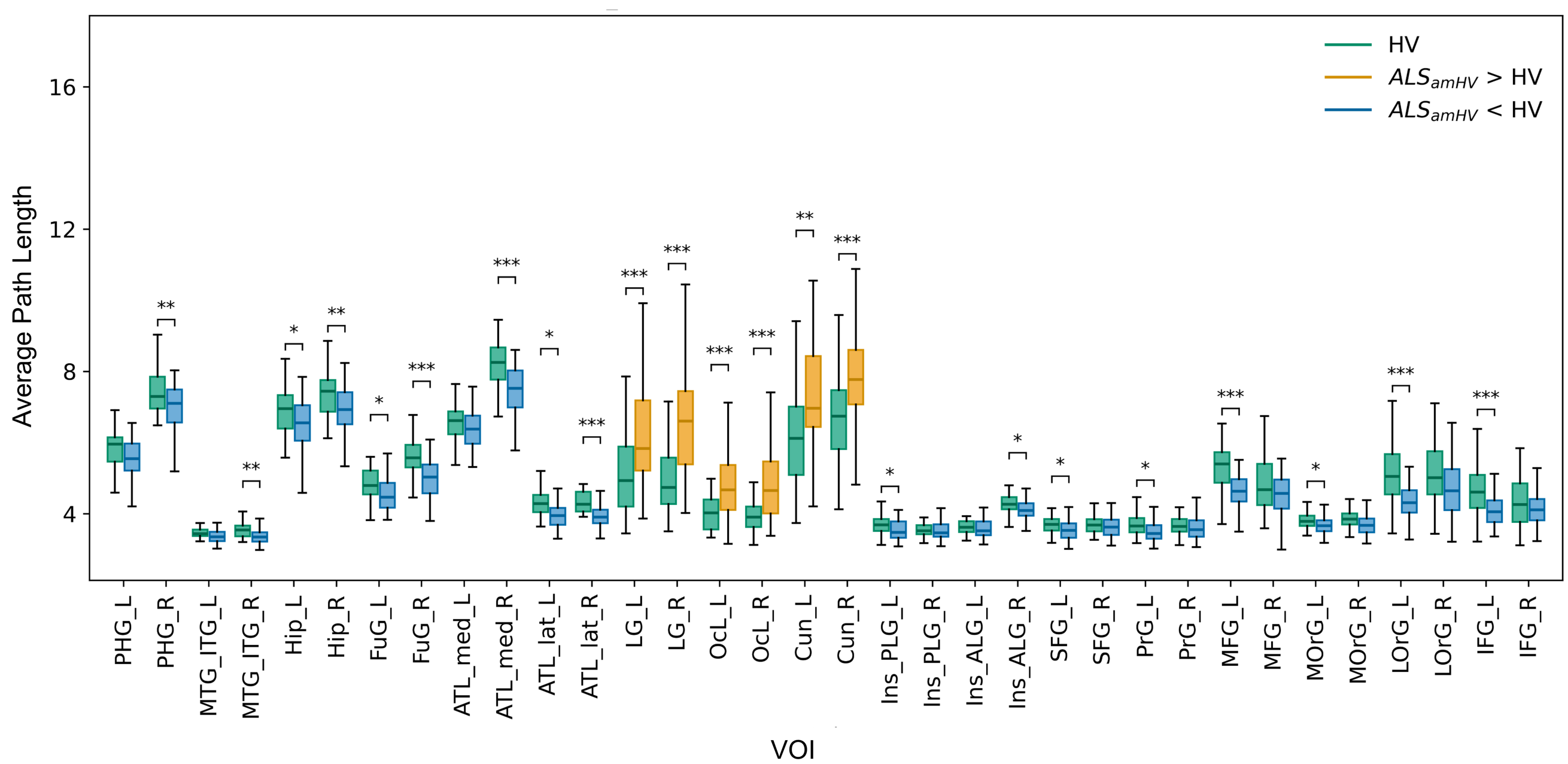

Figure S6: Nodal average path length of the Wasserstein distance (WD)–based connectivity network for the age-matched ALS (ALS_{amHV}) group compared with the HV group. Only regions showing significant group differences in at least one hemisphere are displayed. $ALS_{amHV} > HV$ and $ALS_{amHV} < HV$ indicate regions with significantly higher or lower average path length in ALS_{amHV} compared with healthy volunteers, respectively. Significance levels are indicated as follows: $^{*}p < 0.05$, $^{**}p < 0.01$, $^{***}p < 0.001$

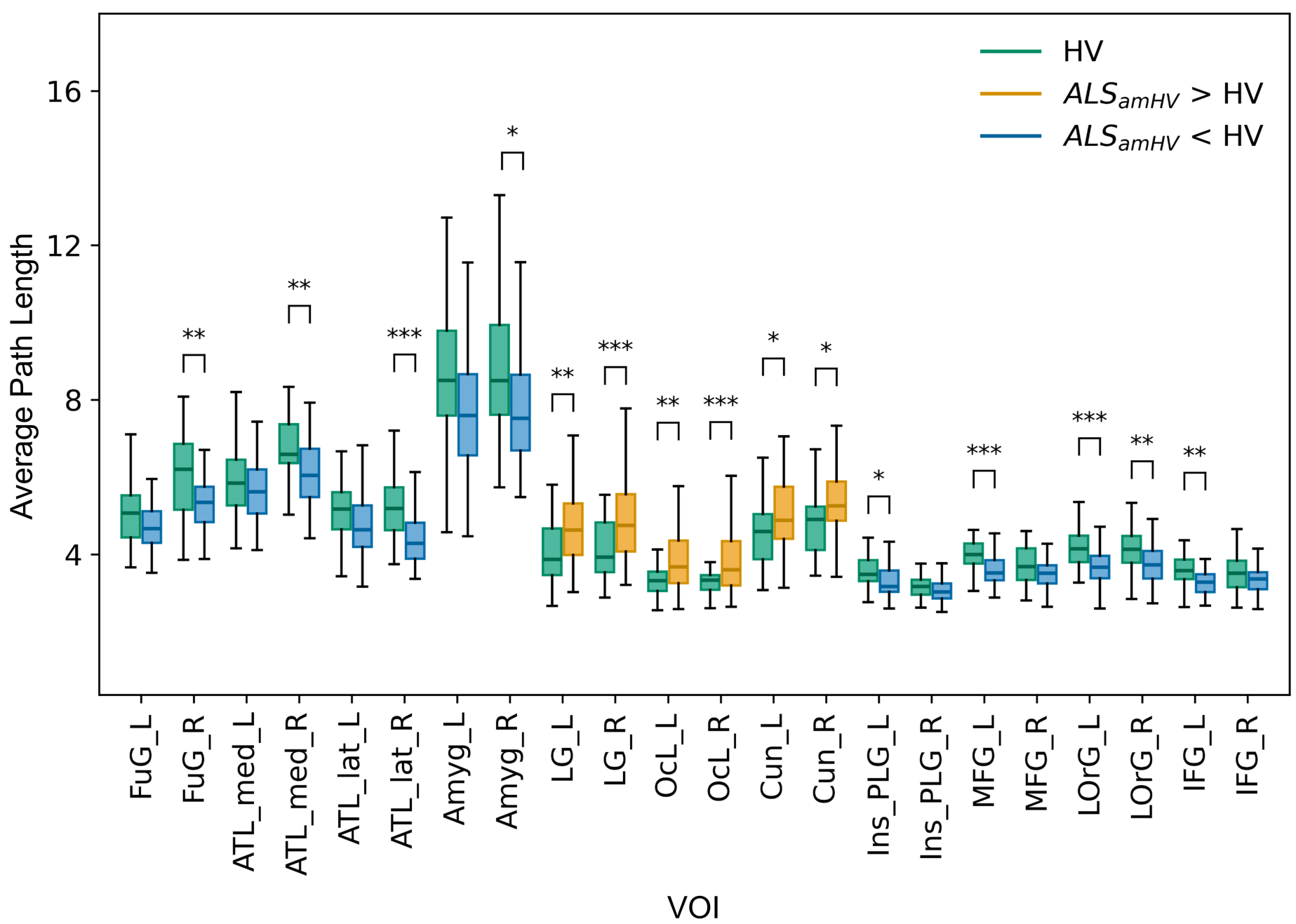

Figure S7: Nodal average path length of the KLSE–based connectivity network for the age-matched ALS (ALS_{amHV}) group compared with the HV group. Only regions showing significant group differences in at least one hemisphere are displayed. $ALS_{amHV} > HV$ and $ALS_{amHV} < HV$ indicate regions with significantly higher or lower average path length in ALS_{amHV} compared with healthy volunteers, respectively. Significance levels are indicated as follows: $^{*}p < 0.05$, $^{**}p < 0.01$, $^{***}p < 0.001$
